# *Cryptosporidium* Pig Genotype II in Immunocompetent Man

**DOI:** 10.3201/eid1506.07621

**Published:** 2009-06

**Authors:** Martin Kváč, Dana Květoňová, Bohumil Sak, Oleg Ditrich

**Affiliations:** Academy of Sciences of the Czech Republic, České Budějovice, Czech Republic (M. Kváč, D. Květoňová, B. Sak, O. Ditrich); University of South Bohemia, České Budějovice (M. Kváč)

**Keywords:** Cryptosporidium, zoonoses, parasites, protozoa, Czech Republic, letter

**To the Editor**: Protozoan parasites from the genus *Cryptosporidium* have been described as a cause of diarrheal disease in immunodeficient and immunocompetent humans worldwide. Although *C. hominis* and *C. parvum* (cattle genotype) cause most infections, humans can be infected by several other *Cryptosporidium* species or genotypes: *C. meleagridis*; *C. felis*; *C. canis*; *C. suis*; *C. muris*; *C. andersoni*; *C. hominis* monkey genotype; *C. parvum* (mouse genotype); and *Cryptosporidium* rabbit genotype, deer genotype, skunk genotype, horse genotype, and chipmunk genotype I (*1*–*4*). Wild and domestic animals are sources of infection for humans (and other animals) and important contributors to contamination of food and drinking water; many nonhuman *Cryptosporidium* species or genotypes are detected in untreated water (*5*). We examined the diversity of *Cryptosporidium* spp. in immunocompetent persons in South Bohemia in the Czech Republic.

Diarrheal fecal samples (n = 457) from 203 anonymous immunocompetent patients <69 years of age with suspected cryptosporidiosis (at least 2 samples/patient/3-day period) were obtained from local health departments and public hospitals in South Bohemia during 2005–2007. Samples were examined for *Cryptosporidium* oocysts by using aniline-carbol-methyl violet staining and light microscopy at × 1,000 magnification (*6*). The microscopically positive samples were confirmed by DNA sequencing of the small subunit (SSU) rRNA gene. Total DNA was extracted from 200–300 mg stool by using the QIAamp DNA Stool Mini Kit (QIAGEN, Hilden, Germany), following the manufacturer’s instructions, after previous homogenization and disruption of oocysts with the Mini-BeadBeater (Biospec Products, Bartlesville, OK, USA). An ≈830-bp fragment of the SSU rRNA gene was amplified by nested PCR according to Jiang et al. (*7*). Purified PCR products were sequenced in both directions on an ABI3130 sequencer analyzer (Applied Biosystems, Foster City, CA, USA) by using the secondary PCR primers and the BigDye Terminator v3.1 Cycle Sequencing Kit (Applied Biosystems). Sequences were assembled by using Chromas Pro (www.technelysium.com.au/chromas.html) and aligned with reference sequences using ClustalX (ftp://ftp-igbmc.u-strasbg.fr/pub/ClustalX). The BLAST server (www.ncbi.nlm.nih.gov/BLAST) was used for DNA database searches. The SSU rRNA gene partial sequences of the 7 patient isolates have been submitted to GenBank (Table).

**Table:** *Cryptosporidium* genotypes identified by using sequencing of partial sequences of the small subunit rRNA gene in the stool samples of immunocompetent humans, Czech Republic

Patient no.	Age, y/sex	Examination year	*Cryptosporidium* species/genotype	Infection intensity*		GenBank accession no.
				Sample 1	Sample 2	
H15	9/M	2005	*C. parvum†*	56	78	EU331237
H23	10/M	2005	*C. hominis*	77	121	EU331242
H98	10/F	2005	*C. parvum†*	43	25	EU331238
H101	11/M	2006	*C. parvum†*	11	5	EU331239
H132	8/M	2006	*C. parvum†*	150	62	EU331240
H158	11/M	2007	*C. parvum†*	26	85	EU331241
H199	29/M	2007	*Cryptosporidium* pig genotype II	38‡	27‡	EU331243

*Numbers of oocysts per 30 fields at ×1,000 magnification, unless otherwise indicated. †Bovine genotype. ‡Numbers of oocysts per whole slide at ×1,000 magnification.

Of the 203 patients, 7 (3.4%) (6 children and 1 adult) had positive results for *Cryptosporidium* spp. Moreover, all samples obtained from these persons during the 3-day period were *Cryptosporidium* spp. positive; partial sequences of the *Cryptosporidium* SSU rRNA gene were obtained from all positive samples identifying 3 different species or genotypes of *Cryptosporidium*. Five were *C. parvum* (bovine genotype), 1 was *C. hominis,* and 1 contained the *Cryptosporidium* pig genotype II (Table). *Cryptosporidium* pig genotype II was found in stool samples from a 29-year-old man who also was infected with *Giardia intestinalis* (assemblage A) (data not shown).

Only *C. parvum* (bovine genotype), *C. hominis*, and *Cryptosporidium* rabbit genotype have been implicated in waterborne outbreaks of cryptosporidiosis in humans. Further studies are needed to determine the potential of other cryptosporidia of animal origin. Recent genetic and biologic characterization studies have identified 2 distinct host-adapted cryptosporidia in pigs, *C. suis* and *Cryptosporidium* pig genotype II. Furthermore, both above-mentioned cryptosporidia have been identified in untreated water (*8*). Pigs could be sources of *Cryptosporidium* water and food pollution and a consequent risk to public health.

Although human infection with *C. suis* has been previously described (*9*), human infection with *Cryptosporidium* pig genotype II has been never reported. This genotype was found in diarrheal stool of 1 adult patient in this study. However, onset of diarrhea could have been caused by co-infection with *G. intestinalis* (assemblage A), which recently also has been described in pigs (*10*). Contact with infected animals and ingestion of contaminated food or water could be the source of both *Cryptosporidium* and *Giardia* infection in the *Cryptosporidium* pig genotype II–positive patient. The passage of oocysts can be excluded because of the number of oocysts detected in repeat samples (Table). Moreover, identification of the infection in an immunocompetent patient underlines the zoonotic potential of this pig genotype and possible presence of risk factors in rural areas with poor water treatment or inadequate biosecurity in pig units. Further evidence of the zoonotic potential of this *Cryptosporidium* genotype is needed to show its pathogenic potential in immunocompetent patients as a cause of gastroenteritis (in the absence of *Giardia* spp. and other established enteropathogens) and to demonstrate invasive tissue stages. The use of molecular techniques to identify *Cryptosporidium* spp. probably will show more zoonotic species or genotypes in humans.

## References

[1] Feltus DC, Giddings CW, Schneck BL, Monson T, Warshauer D, McEvoy JM. Evidence supporting zoonotic transmission of *Cryptosporidium* spp. in Wisconsin. J Clin Microbiol. 2006;44:4303–8. 10.1128/JCM.01067-06 17005736 PMC1698413

[2] Nichols G. Epidemiology. In: Fayer R, Xiao L, editors. *Cryptosporidium* and cryptosporidiosis. Boca Raton (FL): CRC Press; 2007. p. 79–118.

[3] Robinson G, Elwin K, Chalmers RM. Unusual *Cryptosporidium* genotypes in human cases of diarrhea. Emerg Infect Dis. 2008;14:1800–2. 10.3201/eid1411.080239 18976577 PMC2630733

[4] Ajjampur SS, Gladstone BP, Selvapandian D, Muliyil JP, Ward H, Kang G. Molecular and spatial epidemiology of cryptosporidiosis in children in a semiurban community in South India. J Clin Microbiol. 2007;45:915–20. 10.1128/JCM.01590-06 17251402 PMC1829120

[5] Xiao L, Fayer R, Ryan U, Upton SJ. *Cryptosporidium* taxonomy: recent advances and implications for public health. Clin Microbiol Rev. 2004;17:72–97. 10.1128/CMR.17.1.72-97.2004 14726456 PMC321466

[6] Miláček P, Vítovec J. Differential staining of cryptosporidia by aniline-carbol-methyl violet and tartrazine in smears from faeces and scraping of intestinal mucosa. Folia Parasitol (Praha). 1985;32:50. 2580763

[7] Jiang J, Alderisio KA, Xiao L. Distribution of *Cryptosporidium* genotypes in storm event water samples from three watersheds in New York. Appl Environ Microbiol. 2005;71:4446–54. 10.1128/AEM.71.8.4446-4454.2005 16085835 PMC1183313

[8] Ryan U, Read C, Hawkins P, Warnecke M, Swanson P, Griffith M, Genotypes of *Cryptosporidium* from Sydney water catchment areas. J Appl Microbiol. 2005;98:1221–9. 10.1111/j.1365-2672.2005.02562.x 15836492

[9] Xiao L, Bern C, Arrowood M, Sulaman I, Zhou L, Kawai V, Identification of *Cryptosporidium* pig genotype in a human patient. J Infect Dis. 2002;185:1846–8. 10.1086/340841 12085341

[10] Langkjaer RB, Vigre H, Enemark HL, Maddox-Hyttel C. Molecular and phylogenetic characterization of *Cryptosporidium* and *Giardia* from pig and cattle in Denmark. Parasitology. 2007;134:339–50. 10.1017/S0031182006001533 17076923

